# IMP2/IGF2BP2 expression, but not IMP1 and IMP3, predicts poor outcome in patients and high tumor growth rate in xenograft models of gallbladder cancer

**DOI:** 10.18632/oncotarget.21116

**Published:** 2017-09-21

**Authors:** Sonja M. Kessler, Eva Lederer, Stephan Laggai, Nicole Golob-Schwarzl, Kevan Hosseini, Johannes Petzold, Caroline Schweiger, Robert Reihs, Marlen Keil, Jens Hoffmann, Christian Mayr, Tobias Kiesslich, Martin Pichler, Kyung Sik Kim, Hyungjin Rhee, Young Nyun Park, Sigurd Lax, Peter Obrist, Alexandra K. Kiemer, Johannes Haybaeck

**Affiliations:** ^1^ Institute of Pathology, Medical University of Graz, Graz, Austria; ^2^ Department of Pharmacy, Pharmaceutical Biology, Saarland University, Saarbrücken, Germany; ^3^ Center for Biomarker Research in Medicine, Graz, Austria; ^4^ Experimental Pharmacology and Oncology Berlin-Buch GmbH, Berlin, Germany; ^5^ Laboratory for Tumor Biology and Experimental Therapies, Institute of Physiology and Pathophysiology, Department of Internal Medicine I, Paracelsus Medical University, Salzburg, Austria; ^6^ Division of Oncology, Department of Internal Medicine, Medical University of Graz, Graz, Austria; ^7^ Research Unit for Non-coding RNAs and Genome Editing in Cancer, Medical University of Graz, Graz, Austria; ^8^ Department of Experimental Therapeutics, The University of Texas MD Anderson Cancer Center, Houston, USA; ^9^ Department of Surgery, Yonsei University College of Medicine, Seoul, South Korea; ^10^ Department of Radiology, Brain Korea 21 PLUS Project for Medical Science, Yonsei University College of Medicine, Seoul, South Korea; ^11^ Department of Pathology, Brain Korea 21 PLUS Project for Medical Science, Yonsei University College of Medicine, Seoul, South Korea; ^12^ Department of Pathology, General Hospital Graz Sued-West, Graz, Austria; ^13^ Laboratory of Pathology, Dr. Obrist and Dr. Brunhuber OG, Zams, Austria; ^14^ Department of Pathology, Medical Faculty, Otto-von-Guericke-University Magdeburg, Magdeburg, Germany

**Keywords:** IGF2BP2/IMP2/p62, IGF2BP3/IMP3, IGF2BP1/IMP1, NADPH oxidase, gallbladder carcinoma

## Abstract

Overexpression of the oncofetal insulin-like growth factor 2 mRNA-binding protein 2 (IMP2/IGF2BP2) has been described in different cancer types. Gallbladder carcinoma (GBC) is a rare but highly aggressive cancer entity with late clinical detection and poor prognosis.

The aim of this study was to investigate the role of IMP2 in human GBC.

Tissue microarrays (TMAs) of an international multi-center GBC sample collection from *n* = 483 patients were analyzed by immunohistochemistry. IMP2 immunoreactivity was found in 74.3% of the tumor samples on TMA, of which 14.0% showed strong and 86.0% low staining intensity. 72.4% of the tumor samples were IMP1 positive, but IMP1 showed lower expression in tumor tissue compared to control tissues. IMP3 immunoreactivity was observed in 92.7% of all tumors, of which 53.6% revealed strong IMP3 expression. Kaplan-Meier analysis linked high IMP2 expression to shorter survival time (*p* = 0.033), whereas neither IMP1 nor IMP3 expression was linked to a decreased survival time. Eight different human biliary tract cancer (BTC) cell lines were evaluated for tumor growth kinetics in mouse xenografts. Cell lines with high IMP2 expression levels showed the fastest increase in tumor volumes in murine xenografts. Furthermore, *IMP2* expression in these cells correlated with the generation of reactive oxygen species (ROS) and *RAC1* expression in BTC cells, suggesting RAC1-induced ROS generation as a potential mechanism of IMP2-promoted progression of GBC.

In conclusion, IMP2 is frequently overexpressed in GBC and significantly associated with poor prognosis and growth rates *in vivo*. IMP2 might therefore represent a new target for the treatment of advanced GBC.

## INTRODUCTION

Gallbladder carcinoma (GBC) is a lethal malignancy with a 5-year survival depending on tumor stages and being less than 10–20% in advanced tumors. The median survival time for patients with GBC is limited to one year [1]. This is mostly due to late diagnosis, thereby minimizing chances of cure. If diagnosed at early tumor stages (T1 tumors), GBC can be cured in 61–100% [2]. Risk factors of GBC include chronic inflammation, gallstones, female gender, and high age. Complete surgical resection of the tumor is the only curative treatment. For unresectable GBC, chemotherapy is the only approved treatment strategy, but is only applied as a palliative therapeutic route with low efficacy. In spite of multiple studies on potentially attractive new diagnostic and therapeutic targets regarding GBC [3], the translation of these efforts in clinical management of GBC has been very limited so far. As a consequence, the identification and characterization of markers with diagnostic potential and significance is essential to stratify patients into different risk groups for GBC and to develop new therapeutic strategies.

The insulin-like growth factor 2 (*IGF2*) mRNA-binding protein (IMP) IMP2/IGF2BP2 belongs to the family of three IMPs, namely IMP1-3. The IMP family member IMP3 has been shown to be of diagnostic and prognostic relevance in other biliary malignancies, e.g. pancreatobiliary malignancies and cholangiocellular carcinoma (CCC) [4, 5]. In a small study focusing on hepatocellular carcinoma (HCC), one out of two CCC samples showed overexpression of IMP2 [6]. Overexpression of IMP2 or of its splice variant p62 (also known as IMP2-2 or IGF2BP2-2) was also reported for other tumor types, such as HCC, breast, lung, and esophageal cancer [7–13]. Moreover, a tumor-promoting action of IMP2/p62 was described in HCC and glioblastoma (GBM) [9, 14].

Employing a comprehensive tissue microarray (TMA; *n* = 483) of human GBCs as well as a panel of eight biliary tract cancer (BTC) cell lines for xenograft and *in vitro* investigations, we here show that IMP2 expression, but not IMP1 and IMP3 expression correlates with poor prognosis and *in vivo* xenograft tumor growth. RAC1-induced reactive oxygen species (ROS) generation might facilitate IMP2-induced tumor progression.

## RESULTS

### Clinicopathological features

The cohort consisted of 341 female and 141 male patients and one sample with unreported gender. Survival periods between initial diagnosis and death were available for *n* = 397 patients. Tumor grade data were available for 402 patients with 16% of GBCs cases were categorized as grade 1 (G = 1), 44% as grade 2 (G = 2), 39% as grade 3 (G = 3), and 1% as grade 4 (G = 4). pT, pN, and M categories were available for 335, 147, and 128 patients, respectively. 15.5% of the cases were classified as pT = 1, 40% as pT = 2, 35% as pT = 3, 8% as pT = 4, and 1.5% as carcinoma *in situ* (pTis). pN categories were distributed as follows: 55% pN0, 13% pN1, and 13% pN2 patients. 81% of the cases were classified as M0 and 19% as M1. The association between these clinicopathological parameters and survival is shown in Table 1.

**Table 1:** Clinicopathological characteristics of GBC patients

	*n*	survival
Gender		*p*-value
male	107	
female	290	0.78
Grade		
low (G1/2)	233	
high (G3/G4)	151	< 0.001
Tumor stage (pT)		
low (pT1/2)	199	
high (pT3/4)	165	< 0.001
Lymph node metastasis (pN)		
absent	75	
present	71	< 0.001
Metastasis (M)		
absent	99	
present	35	< 0.001
Vascular invasion (V)		
absent	86	
present	33	< 0.001
Lymphovascular invasion (L)		
absent	86	
present	59	< 0.001

### IMP2/p62 expression, but not IMP1 and IMP3, correlates with tumor grade and lymphovascular invasion

**Figure 1:**
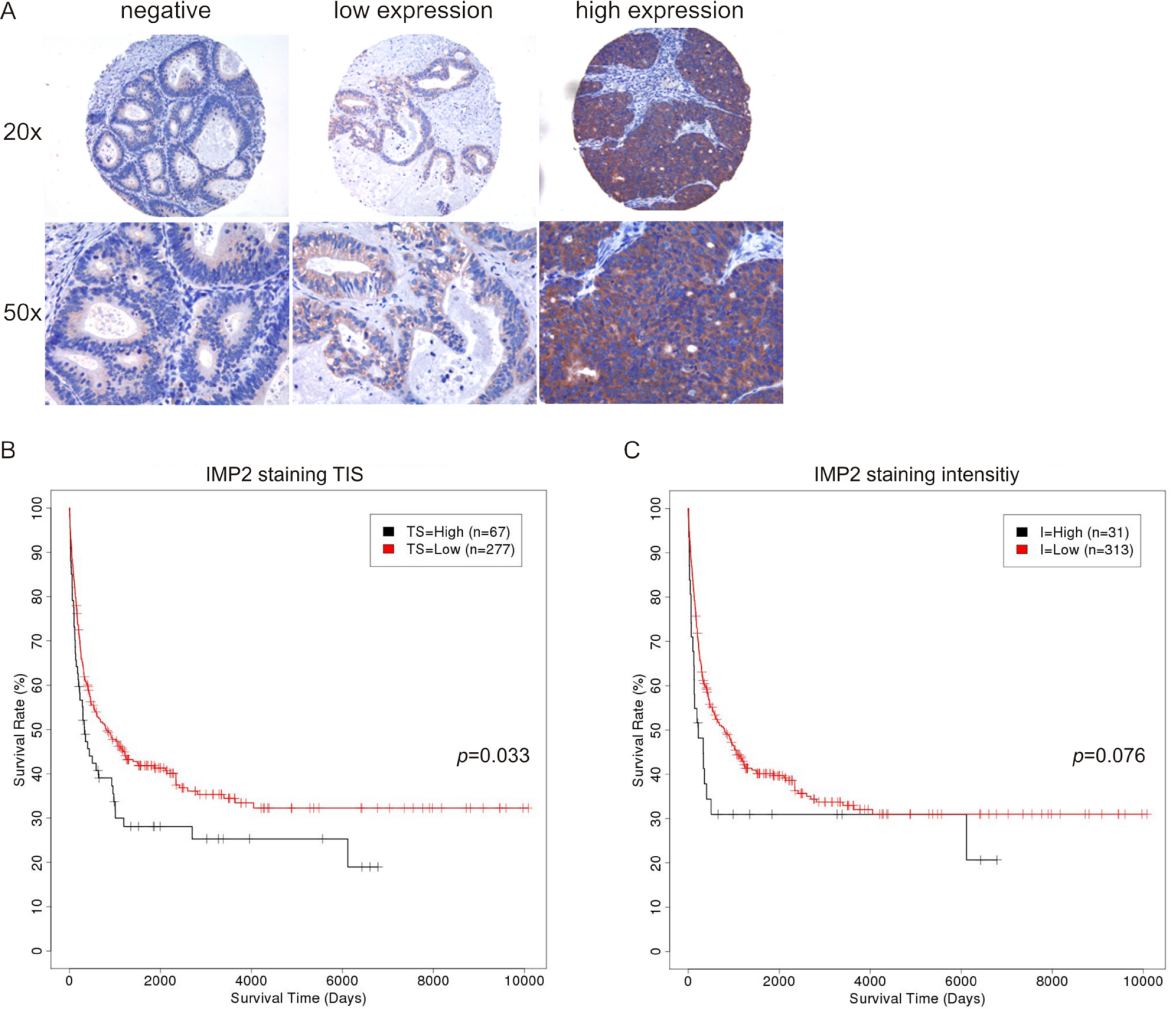
IMP2 expression correlates with short survival and *in vivo* tumor growth (**A**) Representative immunostainings against IMP2 in human GBC. Magnification: 20× and 50×. (**B,** **C**) Kaplan-Meier survival plots referring to low and high IMP2 expression levels in human GBC determined by TIS score (TS) (B) and staining intensity (I) (C).

Immunohistochemical staining revealed an exclusively cytoplasmic localization of IMP2/p62. Nuclear expression of IMP2/p62 was absent in all cases confirming the expected localization [15]. 25.7% of the cases were negative for IMP2/p62 expression, whereas from 74.3% positive samples 86.0% showed low IMP2/p62 expression and 14.0% highly expressed IMP2/p62 (Figure 1A) without being frequently mutated: mutations in the *IGF2BP2* gene were observed in 1.35% of GBC cases investigated (*n* = 74) and in 0% of bile duct cancer samples (*n* = 294) (Sanger Institute COSMIC website, http://www.sanger.ac.uk/cosmic). Interestingly, IMP2/p62 staining intensity as well as total immunostaining (TIS) score correlated with high tumor grade (G3/4) and lymphovascular invasion (L1) (Table 2). A trend for a correlation with higher staining intensity and TIS scores was found without reaching statistical significance for the following parameters: high tumor stage (pT3/4), distant metastasis (M1), and vascular invasion (V1) (Table 2).

**Table 2:** Association between IMP2 protein expression and tumor grades and stages

IMP2	Intensity		TIS			
	low	high	*p*-value	low	high	*p*-value
Grade						
low (G1/2)	195	11		179	27	
high (G3/4)	109	20	0.0018	90	39	< 0.001
Tumor stage (pT)						
low (T1/2)	156	17		141	32	
high (T3/4)	132	11	0.51	111	32	0.39
Lymph node metastasis (pN)						
absent	67	3		60	10	
present	64	4	0.67	60	8	0.66
Metastasis (M)						
absent	91	3		85	9	
present	28	2	0.40	26	4	0.056
Vascular invasion (V)						
absent	79	2		74	7	
present	27	3	0.089	24	6	0.098
Lymphovascular invasion (L)						
absent	79	2		74	7	
present	48	6	0.037	43	11	0.0495

72.4% of the tumor samples were IMP1 positive, however, IMP1 showed lower expression in tumor tissue compared to control tissues (*p <* 0.001) (Supplementary Figure 1A). IMP1 staining intensity inversely correlated with tumor grading (Supplementary Table 1). Both staining intensity and TIS score of IMP1 immunostaining negatively correlated with the presence of lymph node metastasis (N1), distant metastasis (M1), vascular invasion (V1), and lymphovascular invasion (L1) (Supplementary Table 1).

IMP3 immunoreactivity was observed in 92.7% of all tumors, of which 53.6% revealed strong IMP3 expression (Supplementary Figure 2A). IMP3 did not correlate with any of the investigated clinicopathological parameters (Supplementary Table 2).

### High IMP2/p62 expression is linked to short survival and p62 enhances tumor growth *in vivo*

Kaplan-Meier analysis of high versus low IMP2/p62 expressing samples revealed a significantly reduced survival in patients with high TIS score (*p* = 0.033; Figure 1B). Furthermore, high staining intensity tended to be linked to short survival (*p* = 0.076; Figure 1C). Staining intensity of IMP1 expression (*p* = 0.019; Supplementary Figure 1B) but not TIS score (*p* = 0.122; Supplementary Figure 1C) correlated with longer survival time. IMP3 expression was not linked to survival time (Supplementary Figure 2B, 2C).

**Figure 2:**
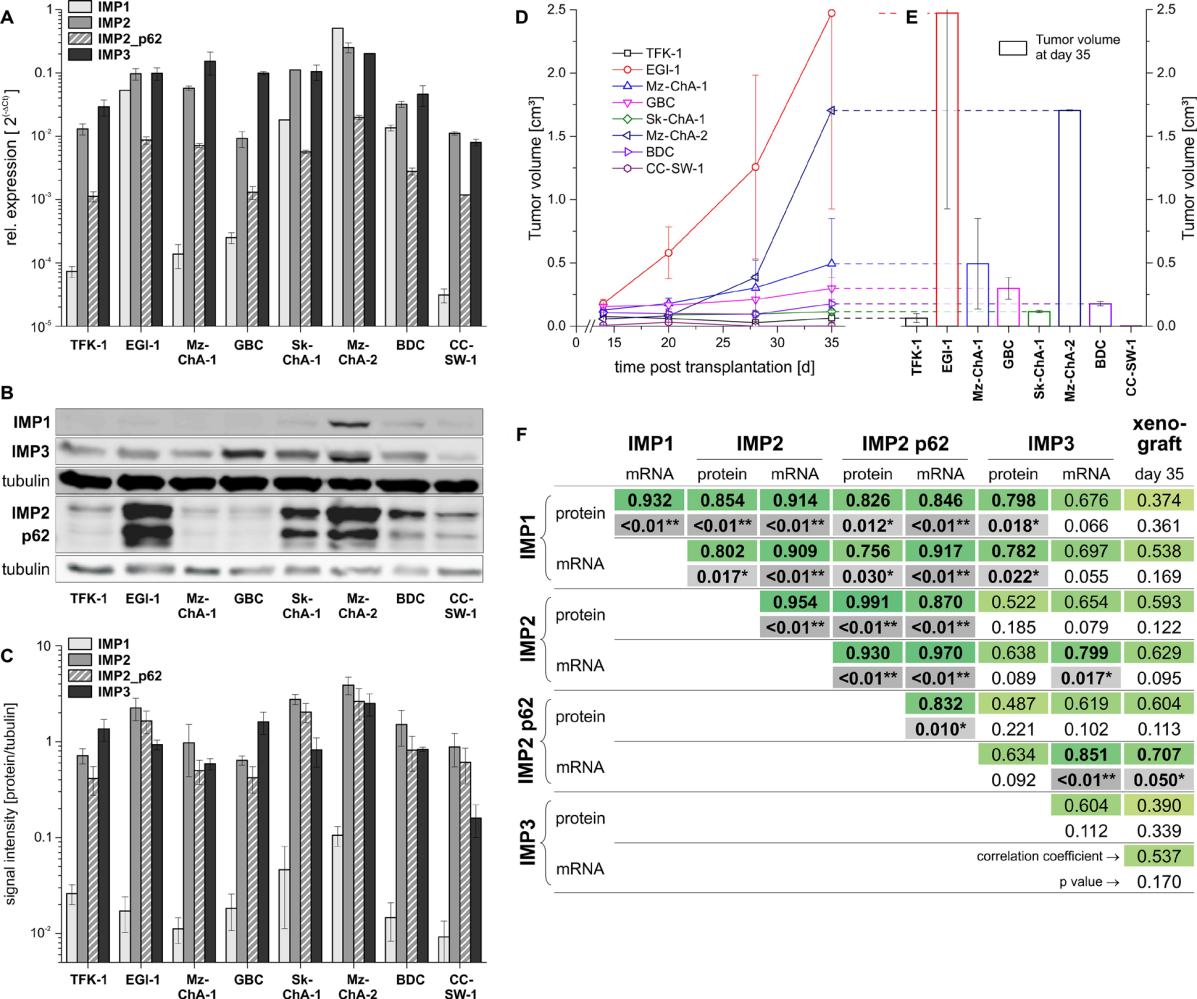
IMP2 expression is linked to *in vivo* tumor growth (**A**) Expression of IMP1, IMP2 and its splice variant p62 as well as IMP3 in eight different BTC cell lines analyzed by qPCR (A) and Western blot (**B**, **C**). (B) Representative Western blot showing IMP1, IMP2, p62, and IMP3 expression. (C) Signal intensities normalized to tubulin are shown (*n* = 3, 1–3 replicates). (**D**, **E**) Tumor growth of murine xenografts of the respective human BTC cell line (*n* = 2 each). Data are shown as mean ± SEM. (**F**) Correlation analysis between IMP2/p62 expression and tumor growth.

In order to investigate the effect of IMPs on tumor formation we employed a murine xenograft model with eight different BTC cell lines: five bile duct cancer cell lines [CC-SW-1 (Grade 2), BDC (Grade 4), EGI-1 (Grade 3), Sk-ChA-1 (Grade 3), TFK-1 (Grade 2)] and three gallbladder cancer cell lines [Mz-ChA-1 (Grade 1), Mz-ChA-2 (Grade 2), GBC (Grade 1)] [16]. Seven out of the eight BTC cell lines were tumorigenic, while CC-SW-1 cells did not grow at all in nude mice. Of note, IMP2 expression and expression of its splice variant p62 were highest in cell lines originating from less differentiated metastases or metastasizing primary tumors: EGI-1, Mz-ChA-2, and Sk-ChA-1 [17, 18] (Figure 2A–2C). EGI-1 and Mz-ChA-2, both cell lines highly expressing *IMP2* and *p62* (Figure 2A), showed the fastest tumor growth *in vivo* (Figure 2D, 2E). Tumor growth significantly correlated only with *p62* expression (Figure 2F). Correlation with cell cycle data [19] revealed that IMP2/p62 expression in the BTC cell lines positively correlated with cells in the S, G2, and SG2 phase, whereas a negative correlation was found with cells in the G0/G1 phase (data not shown). Interestingly, also two excised, patient-derived cholangiocarcinoma xenografts were analyzed and revealed strong IMP2 and p62 expression and a distinct tumor growth (Supplementary Figure 3). Neither tumor expressed IMP1 and only one of the tumors expressed IMP3 (Supplementary Figure 3A).

### IMP2 and p62 expression are associated with *RAC1* expression and ROS generation

**Figure 3:**
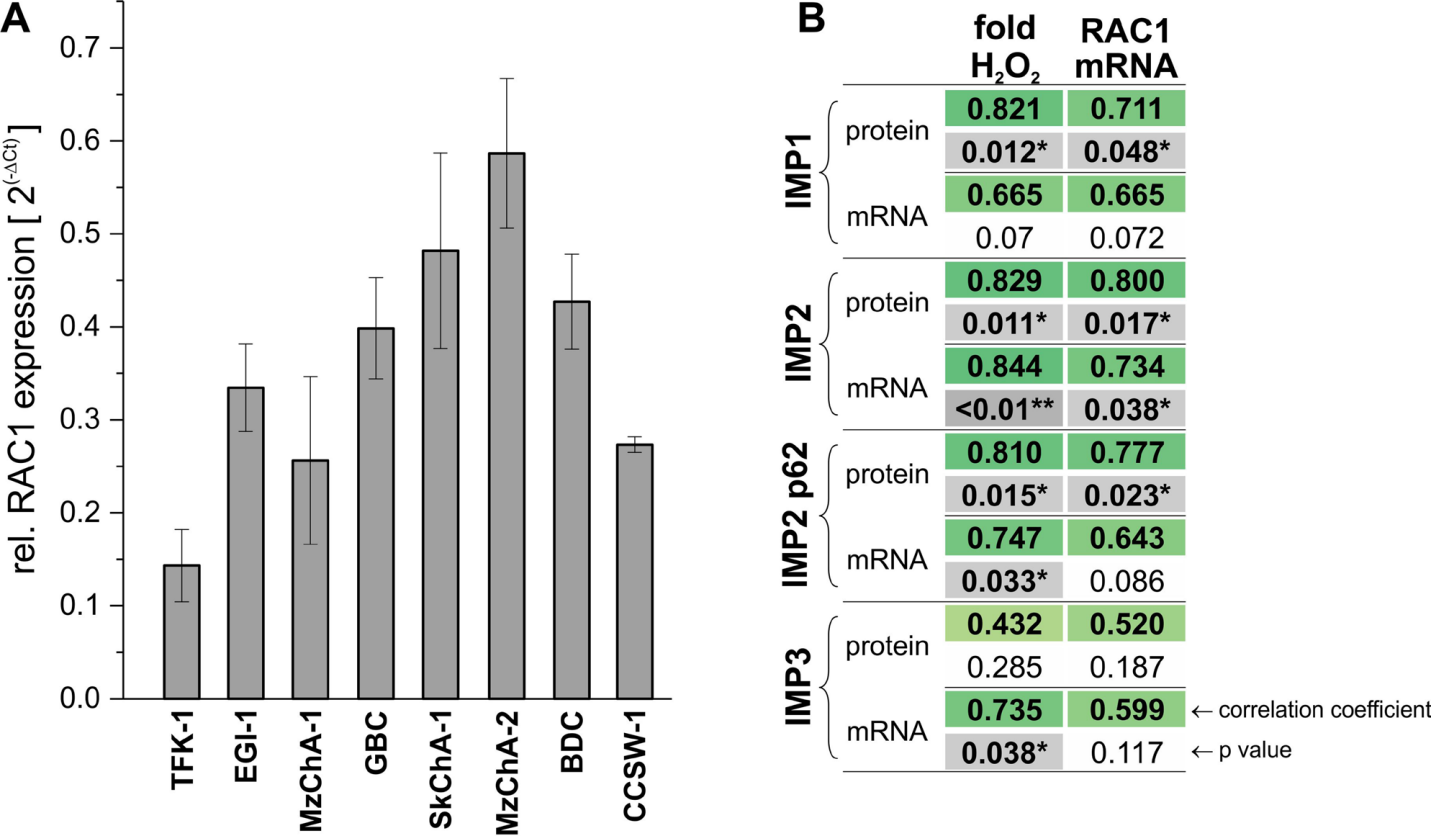
Correlation of IMP2 expression with *RAC1* expression and ROS generation (**A**) Expression of *RAC1* in eight different BTC cell lines analyzed by qPCR. (**B**) Correlation analysis between IMP expression and ROS levels and *RAC1* mRNA levels. ROS levels (fold H_2_O_2_) are displayed as fold change of PMA-stimulated cells compared to untreated control.

Since p62 has been described to induce a more advanced tumor phenotype by promoting RAC1-induced ROS generation [9], *RAC1* mRNA expression was measured in the eight BTC cell lines. We observed that *RAC1* levels highly correlated with *IMP2* mRNA and IMP2 protein as well as p62 protein levels (Figure 3A, 3B). Of note, IMP1 protein levels also correlated with RAC1 expression. In order to test the significance of IMP2/p62-RAC1 correlation ROS levels were measured in the BTC cell lines. IMP2 and p62 mRNA and protein expression in the BTC cell lines were linked to an increased ROS production (Figure 3A, 3B). Also IMP1 protein and *IMP3* RNA expression were associated with increased ROS production.

## DISCUSSION

A splice variant of the *IMP2* gene, p62/IMP2-2, was originally identified as a tumor-associated auto-antigen with auto-antibodies against p62/IMP2-2 detected in HCC patients [13] and in several other cancer types [11, 12, 20]. Recently, *IMP2* knockout mice were suggested to exhibit resistance towards malignancy [21], while *p62/IMP2-2* transgenic mice were reported to be more prone to hepatocarcinogenesis showing a more aggressive HCC phenotype [9]. Despite one study presenting IMP2 expression in one out of two investigated cases of CCC [6], nothing is known about the role of IMP2 in biliary tract cancers. To the best of our knowledge, this is the first study based on a sufficient sample size, which correlates IMP2/p62 expression with tumor progression or patient survival in GBC.

In around 80% of GBC patients their cancer has infiltrated adjacent organs or has spread to distant sites at the time of diagnosis. This is due to the fact that early symptoms are rather non-specific, and effective biomarkers for GBC diagnosis are lacking. The same is true for prognostic markers of GBC progression. Although the prognostic relevance of several tissue biomarkers has been studied, none of them has made it as routine screening into the clinics so far [22].

Our immunohistochemical analysis of the largest human GBC collection we are aware of clearly showed that high IMP2/p62 expression is associated with advanced tumors and short survival. However, IMP3, which has been suggested as a diagnostic marker in GBC [23], did not correlate with prognosis in our study. In contrast, although IMP1 is known to be overexpressed and to promote tumor growth in HCC [24], IMP1 expression was associated with a rather better prognosis in GBC. IMP2/p62 expression has been reported to correlate with poor prognosis in other cancer entities such as HCC, glioblastoma (GBM), head and neck cancer, breast, and esophageal cancer [7, 8, 10, 14, 25]. Similarly, *in vitro* IMP2 expression was strongest in BTC cells originating from adenocarcinoma metastasis or metastasizing primary adenocarcinomas characterized by low differentiation underlining the correlation with poor outcome. The enhanced tumor growth of xenografts of cell lines highly expressing IMP2 suggests a tumor-promoting action of IMP2 in GBC. In hepatocarcinogenesis, IMP2 has been shown to facilitate tumor development [9], underlining the results of our study.

However, it is not clear how upregulation or aberrant expression of IMP2/p62 contributes to the neoplastic process in GBC. IMP2/p62 has been shown to induce ROS generation by an enhanced activation of the small GTPase RAC1 due to increased expression of the paracrine factor DLK1 [9]. The correlation of IMP2 with *RAC1* and ROS levels suggests the existence of a similar mechanism in GBC. RAC1 itself has been linked to poor prognosis and short survival in GBC [26]. Another pathway that might involve miR-1275, which has been described to target IMP2 [27] and is downregulated in GBC [28]. In GBMs IMP2 was shown to regulate oxidative phosphorylation, thereby affecting clonogenicity and tumorigenicity [14]. Furthermore, IMP2 was suggested to contribute to breast cancer progression by increasing cell migration and reducing cell adhesion to extracellular matrix proteins [29]. This is in line with the correlation of IMP2 and RAC1 expression, since RAC1 is also an important factor for cell migration [30, 31]. Still, further studies will be needed to shed light on the exact mechanisms by which IMP2/p62 is able to promote GBC progression.

In summary, this study shows for the first time a correlation between patient survival as well as tumor progression and IMP2/p62 expression in GBC, which might involve RAC1-induced ROS production.

## MATERIALS AND METHODS

### Cohort for TMA

Formalin-fixed, paraffin-embedded gallbladder tissue samples and the corresponding clinical data were collected for 483 GBC patients from the Medical University of Innsbruck (12%), University Hospital of Seoul (23%), General Hospital Graz West (7%), and the Medical University of Graz (58%). The tissue samples were used for generating TMAs for further analyses. Due to the limited amount of tissue, staining of all samples could only be realized for IMP2/p62. For IMP1 and IMP3, a lower sample number was used for exploratory analyses (*n* = 350 and *n* = 179, respectively).

Samples and data from the University Hospital of Graz and the General Hospital Graz South West used in this project were provided by the Biobank Graz under the permission of the ethics commission (EK number 24-177 ex 11/12). In addition, authorization was given by the institutional review board of the Severance Hospital (no. 4-2014-0421), and the requirement for informed consent was waived. A small part of gallbladder cancer samples (the study cohort from the University Hospital Innsbruck) was already utilized in a previous study [32] for which it was approved by the institutional review board.

All corresponding haematoxylin/eosin (H/E) slides were centrally reviewed for confirmation of diagnosis and adequacy of material by an experienced and board certified pathologist (JH).

### Immunohistochemistry

Immunohistochemical stainings were performed using the Dako Envision AEC Kit (#K4009, Dako, Germany) for antibody detection according to the manufacturer's instructions. Anti-IMP1 antibody (Santa Cruz), anti-IMP2/p62 antibody [10] detecting both isoforms, and anti-IMP3 antibody (Proteintech) were incubated for 1 h at room temperature.

Immunohistochemistry (IHC) was analyzed by two independent investigators (JH and SMK) by light microscopy. The density and intensity of each core was semi-quantitatively scored (intensity 0 = no detectable staining, 1 = weak staining, 2 = moderate staining, and 3 = strong staining) and differentiated in nuclear and cytoplasmic staining. Density was scaled as 0 for no IHC staining, 1 < 10%, 2 = 10-49%, 3 = 50-80%, and 4 > 80%. For analyzing the data total immunostaining scores (TIS) were calculated by multiplying density and intensity, and the groups were summarized in no staining (0), low staining (1), and high staining (2 and 3).

Non-tumor gallbladder tissue (*n* = 38) and secondary antibody only control were used as controls in immunohistochemistry.

### Homovanillic acid assay

H_2_O_2_ levels were measured using the homovanillic acid (HVA) assay as described previously [33]. 250,000 cells per well were seeded in a 6-well plate. After 24 h cells were washed two times with 1xPBS and freshly prepared HVA solution (100 μM HVA, 4 U/ml horse-radish peroxidase, dissolved in PBS containing Ca^2+^ and Mg^2+^, all from Sigma Aldrich) was added. In order to stimulate H_2_O_2_ production cells were treated with 0.1 μM phorbol 12-myristate 13-acetate (PMA) (Calbiochem) for 5 min before the HVA solution was added. Cells were then incubated for 2 h at 37°C. 40 μl HVA stop buffer (0.1 M glycine, 0.1 M NaOH, 25 mM EDTA in water, all from Sigma Alderich) were added to each well of a black 96-well plate, and 260 μl of the extracellular HVA supernatant were added. The fluorescence (312 nm excitation, 420 nm emission) was then determined using a SpectraMax M5e (Molecular Devices). For the determination of the H_2_O_2_ concentration, a dilution series of H_2_O_2_ (0-5 μM) was treated the same way as described above. For protein quantification a BCA assay was performed.

### Gene expression analysis

Total RNA was isolated from untreated cells 24 h after seeding in 3 cm petri dishes using TRIzol Reagent (Life Technologies, Vienna, Austria) and Direct-zol^TM^ RNA MiniPrep Kit (Zymo Research, Irvine, California, USA) according to the manufacturer's instructions. cDNA synthesis was performed with 1.0 μg total RNA per sample using the GoScript^TM^ Reverse Transcription System (Promega, Mannheim, Germany) and gene expression was analyzed using GoTaq qPCR Master Mix (Promega) on a ViiA7 real-time PCR system (Applied Biosystems, Life Technologies). All samples were measured in biological triplicates and technical quadruplicates within the PCR runs. Melting curve analysis was done to ensure the specificity of primers. Ct values were normalized to the housekeeping genes beta-actin (actb) and beta-2-microglobulin (b2m). KiCqStart™ Primers for *IMP1*, *IMP2* (full length; IGF2BP2_3) and *IMP2-2/p62* (IGF2BP2_1), *IMP3, RAC1* as well as the housekeeping genes were purchased from Sigma Aldrich (Vienna, Austria). Primer sequences were the following: ACTB_fwd: GACGACATGGAGAAAATCTG; ACTB_rev: ATGATCTGGGTCATCTTCTC; B2M_fwd: AAGGACTGGTCTTTCTATCTC; B2M_rev: GATCCCACTTAACTATCTTGG; IGF2BP3_1_fwd: GGAGGAGATCATGAAGAAAATC; IGF2BP3_1_rev: TTTCTGATTGCTCAAACTGC; IGF2BP2_1_fwd: CATATACAACCCGGAAAGAAC; IGF2BP2_1_rev: CTCTGGATAAGAGTGATGATG; IGF2BP2_3_fw: CGGGTAGATATCCATAGAAAAG; IGF2BP2_3_rev: GAATCTCTTCGGCTAGTTTG; IGF2BP1_3_fwd: AGATAGACGTGCATAGGAAG; IGF2BP1_3_rev: GTGTCCTTAGCCTCTTTATG; RAC1_fwd: TTGGTGCTGTAAAATACCTG; RAC1_rev: GGCATTTTCTCTTCCTCTTC.

### Western blot

Western blots were performed as previously described [10]. Antibodies used were specific to IMP1 (Santa Cruz), IMP2/p62 [6, 10] detecting both isoforms, IMP3 (Proteintech), and α-tubulin (#T9026, Sigma, Germany). IRDye680-conjugated anti-rabbit IgG (LiCor Bioscience, Germany) and IRDye800-conjugated anti-mouse IgG (LiCor) were used as secondary antibodies. Signal intensities were determined by using the Odyssey infrared imaging system (LiCor).

### Xenograft models

Eight different human BTC cell lines were tested for tumorigenicity in immunodeficient NMRI nude mice (TFK-1: metastasized extrahepatic biliary adenocarcinoma, EGI-1: extrahepatic biliary adenocarcinoma, Mz-ChA-1: gallbladder adenocarcinoma abdominal wall metastasis, Mz-ChA-2: gallbladder adenocarcinoma liver metastasis, Sk-ChA-1: malignant ascites in extrahepatic biliary adenocarcinoma, BDC: bile duct cancer, CC-SW-1: intrahepatic biliary adenocarcinoma, GBC: gallbladder mucosal carcinoma [19]). Tumor cells (10^7^ cells in 50 μl medium mixed with 50 μL Matrigel™ Basement Membrane Matrix (Dulbecco)) were injected subcutaneously into the left flank of two NMRI nude mice (Taconic, Bomholdtgard, Denmark) per cell line, respectively. Cells were cultured in Dulbecco›s Modified Eagle Medium (DMEM) supplemented with 10% fetal calf serum in 5% CO_2_ at 37°C. All animal procedures were performed in accordance with the local animal welfare committee (Gen0030/15).

Tumor size (length x width) was measured by calipers once weekly for up to 125 days. Individual tumor volumes (V) were calculated by the formula: V = (length x [width]^2^)/2.

### Statistics

For statistical evaluations, the immunohistochemical staining intensities were grouped as low (scores 0–1) and high (scores 2–3). Associations between binary categories were analyzed using the Chi^2^-test.

The data were stored in a relational scheme in the PostgreSQL 9.1 database management system. R and R-Studio with the R PostgreSQL package were used for calculations, Kaplan-Meier survival estimates were generated by the R Survival Package. The likelihood ratio test in Cox regression analysis was used to calculate *p*-values. In case of ties, the Breslow method was applied.

Correlation analysis between expression levels of IMPs and xenograft tumor growth characteristics, *RAC1*, and ROS levels were performed using Pearson's correlation and IBM SPSS v21 (Vienna, Austria).

## SUPPLEMENTARY MATERIALS FIGURES AND TABLES


