# Supplementary material for: IMP2/IGF2BP2 expression, but not IMP1 and IMP3, predicts poor outcome in patients and high tumor growth rate in xenograft models of gallbladder cancer
Source: Oncotarget. 2017 Sep 21;8(52):89736–45. doi: 10.18632/oncotarget.21116 (PMC5685705; doi:10.18632/oncotarget.21116)
Supplement: Supplementary file 1 [file oncotarget-08-89736-s001.pdf]

IMP2/IGF2BP2 expression, but not IMP1 and IMP3, predicts poor outcome in patients and high tumor growth rate in xenograft models of gallbladder cancer

SUPPLEMENTARY MATERIALS

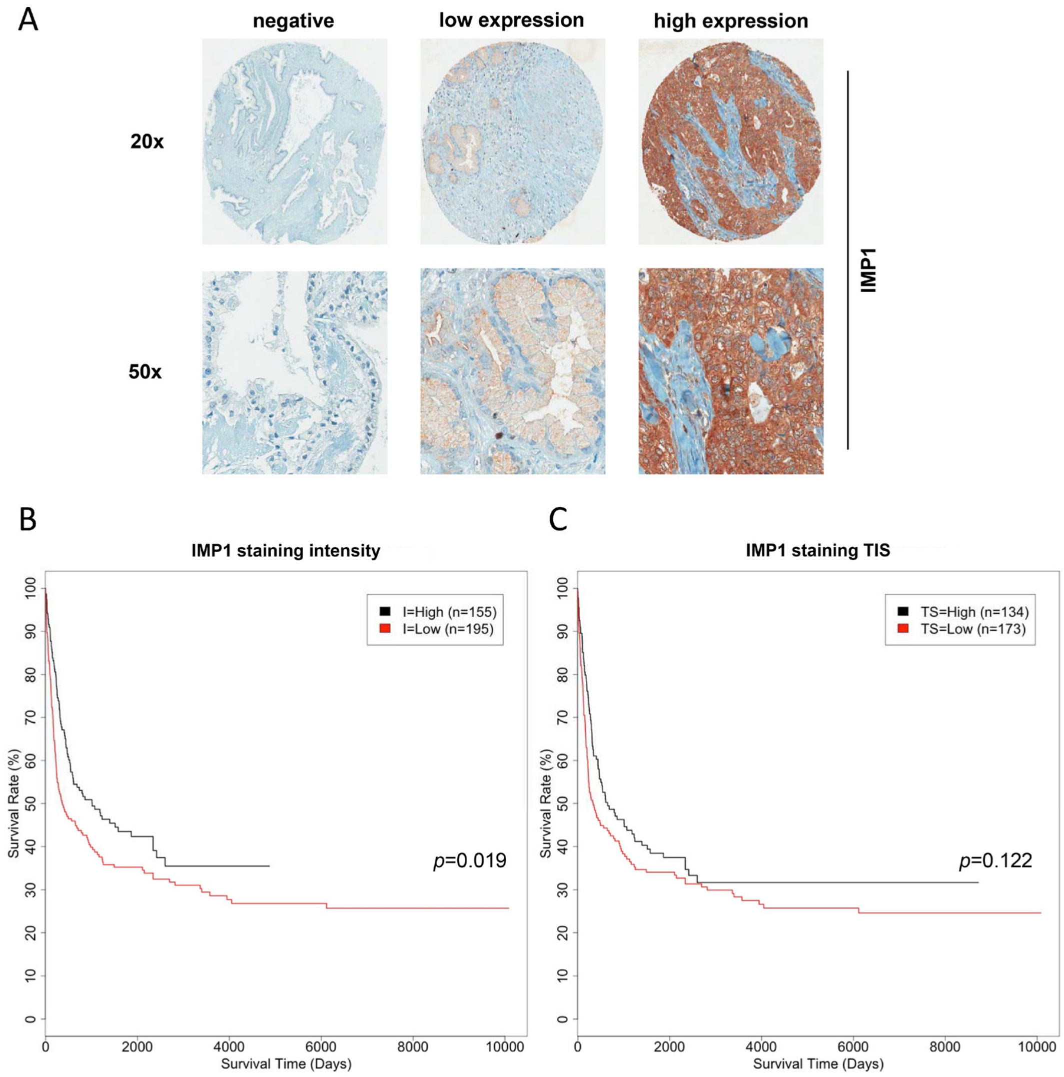

Supplementary Figure 1: IMP1 expression correlates with improved survival. (**A**) Representative immunostainings against IMP1 in human GBC. Magnification: 20× and 50×. (**B**, **C**) Kaplan-Meier survival plots referring to low and high IMP1 expression levels in human GBC determined by staining intensity (I) (B) and TIS score (TS) (C).

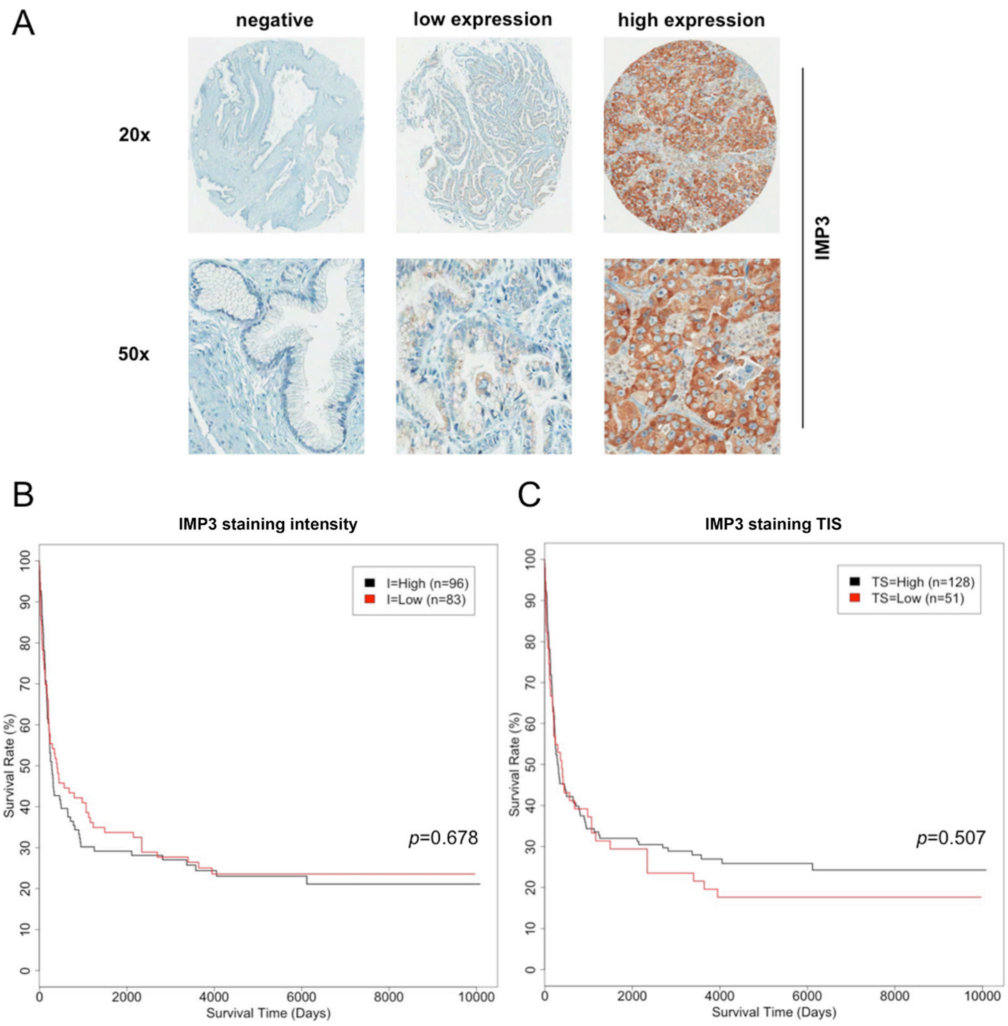

Supplementary Figure 2: IMP3 expression is not linked to survival time. (**A**) Representative immunostainings against IMP3 in human GBC. Magnification: 20× and 50×. (**B**, **C**) Kaplan-Meier survival plots referring to low and high IMP3 expression levels in human GBC determined by staining intensity (I) (B) and TIS score (TS) (C).

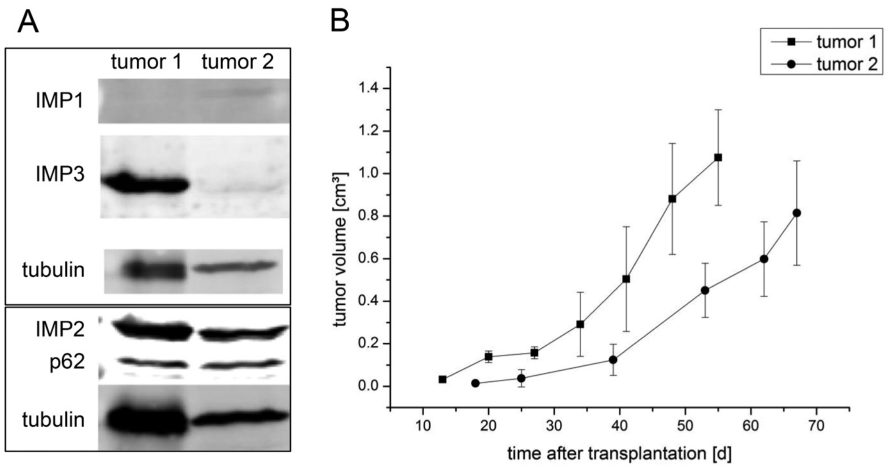

Supplementary Figure 3: IMP expression in excised tumor xenografts and *in vivo* tumor growth. (**A**) Expression of IMP1, IMP2 and its splice variant p62 as well as IMP3 in two different patient-derived, excised cholangiocarcinoma tumor xenografts analyzed by Western blot. (**B**) Tumor growth of murine xenografts of the respective excised tumors. Data are shown as mean ± SEM.

Supplementary Table 1: Association between IMP1 protein expression and tumor grades and stages

IMP1	Intensity			TIS		
	low	high	*p*-value	low	high	*p*-value
Grade						
low (G1/2)	106	108		95	86	
high (G3/4)	83	43	0.003	76	43	0.051
Tumor stage (pT)						
low (T1/2)	90	88		79	75	
high (T3/4)	79	64	0.40	70	54	0.39
Lymph node metastasis (pN)						
absent	15	54		13	31	
present	27	34	0.006	27	23	0.017
Distant metastasis (M)						
absent	4	87		1	55	
present	24	8	< 0.001	23	5	< 0.001
Vascular invasion (V)						
absent	4	74		1	47	
present	10	22	< 0.001	9	13	< 0.001
Lymphovascular invasion (L)						
absent	4	74		1	47	
present	33	23	< 0.001	29	17	< 0.001

Supplementary Table 2: Association between IMP3 protein expression and tumor grades and stages

IMP3	Intensity			TIS		
	low	high	*p*-value	low	high	*p*-value
Grade						
low (G1/2)	47	48		31	64	
high (G3/4)	36	48	0.38	20	64	0.19
Tumor stage (pT)						
low (T1/2)	38	39		22	55	
high (T3/4)	34	42	0.57	23	53	0.82
Lymph node metastasis (pN)						
absent	7	7		4	10	
present	13	16	0.75	8	21	0.94
Distant metastasis (M)						
absent	na	na		na	na	
present	7	15		6	16	
Vascular invasion (V)						
absent	na	na		na	na	
present	4	7		3	8	
Lymphovascular invasion (L)						
absent	na	na		na	na	
present	9	21		6	24	

na: not applicable.
